# Peptide-based PET imaging agent of tumor TIGIT expression

**DOI:** 10.1186/s13550-023-00982-7

**Published:** 2023-05-02

**Authors:** Dinghu Weng, Rong Guo, Ziyang Zhu, Yu Gao, Rui An, Xiuman Zhou

**Affiliations:** 1 Department of Radiology, Zhongnan Hospital of Wuhan University, Wuhan, 430071 Hubei China; 2 Department of Nuclear Medicine, Union Hospital, Tongji Medical College, Huazhong University of Science and Technology, Wuhan, 430000 Hubei China; 3 Hubei Province Key Laboratory of Molecular Imaging, Wuhan, 430000 Hubei China; 4 School of Pharmaceutical Sciences (Shenzhen), SunYat-Sen University, Shenzhen, 518107 Guangdong China

**Keywords:** Immune checkpoint, TIGIT, ^68^Ga, Peptide, Radiotracer, Immunotherapy

## Abstract

**Background:**

Accumulating studies have demonstrated that elevated TIGIT expression in tumor microenvironment correlates with better therapeutic response to TIGIT-based immunotherapy in pre-clinical studies. Therefore, a non-invasive method to detect tumor TIGIT expression is crucial to predict the therapeutic effect.

**Methods:**

In this study, a peptide-based PET imaging agent, ^68^Ga-DOTA-^D^TBP-3, was developed to non-invasively detect TIGIT expression by micro-PET in tumor-bearing BALB/c mice. ^D^TBP-3, a D-peptide comprising of 12 amino acids, was radiolabeled with ^68^Ga through a DOTA chelator. In vitro studies were performed to evaluate the affinity of ^68^Ga-DOTA-^D^TBP-3 to TIGIT and its stability in fetal bovine serum. In vivo studies were assessed by micro-PET, biodistribution, and immunohistochemistry on tumor-bearing BALB/c mice.

**Results:**

The in vitro studies showed the equilibrium dissociation constant of ^68^Ga-DOTA-^D^TBP-3 for TIGIT was 84.21 nM and its radiochemistry purity was 89.24 ± 1.82% in FBS at 4 h in room temperature. The results of micro-PET, biodistribution and immunohistochemistry studies indicated that ^68^Ga-DOTA-^D^TBP-3 could be specifically targeted in 4T1 tumor-bearing mice, with a highest uptake at 0.5 h.

**Conclusion:**

^68^Ga-DOTA-^D^TBP-3 holds potential for non-invasively detect tumor TIGIT expression and for timely assessment of the therapeutic effect of immune checkpoint blockade.

## Introduction

Cancer cells utilize multiple pathways to evade immune-mediated recognition, one of powerful mechanism is the activation of immune checkpoint. Following continuous antigen stimulation, T cells become exhausted and upregulate the immune checkpoint molecules including CTLA-4 and PD-1, which severely limit the survival and function of T cells [1]. At the same time, ligands of these immune checkpoints receptors are overexpressed on antigen-presenting cells and cancer cells in the tumor microenvironment (TME). Over the last decade, antibody-based immunotherapy targeting immune checkpoints proteins like CTLA-4 and PD-1 have been remarkably successful, with obvious therapeutic effects in more than 15 types of advanced human malignancies [2–4]. Despite the success of immune checkpoint blockade therapies, only a small proportion of patients (approximately 20%) benefit from the current available immunotherapies [5]. Therefore, it is essential to discover novel immune checkpoints with high anti-tumor efficacy for various malignancies.

In addition to CTLA-4 and PD-1, TIGIT is an immune checkpoint expressed on CD4^+^ Th cells, CD8^+^ cytotoxic T lymphocytes, FOXP3^+^ regulatory T cells, and NK cells that is a potential target for future cancer immunotherapy [6–8]. TIGIT has two ligands, CD155 (PVR) and CD112 (PVRL2, nectin-2), which are expressed by antigen-presenting cells and tumor cells in TME to decrease the activity of T cells and NK cells. Studies have indicated that elevated TIGIT expression in the TME may be a marker of poor prognosis and that TIGIT^+^CD8^+^ T cells could be an independent prognostic indicator for TIGIT-based immunotherapy and a potential biomarker for responsiveness [9–12]. Furthermore, it has been demonstrated that TIGIT blockade, especially combined with other immune checkpoint inhibitors, may protect against various hematological and solid tumors, and several antibody-based immunotherapies that block the inhibitory activity of TIGIT have been developed [13, 14]. Several clinical trials are currently being developed to evaluate the effect of anti-TIGIT therapies. Therefore, it is necessary to analyze TIGIT expression in tumors before treatment to screen for the population that will benefit from immunotherapy and avoid the unnecessary financial burden for patients who will not respond.

Several approaches including immunohistochemistry (IHC), Western blotting, and PCR have been used to analyze TIGIT expression by various types, including follicular lymphoma, Hodgkin’s lymphoma, small cell lung cancer, hepatocellular carcinoma, squamous cell cancers, renal cell carcinoma, and melanoma [15–21]. IHC is the most commonly used method to detect tumoral TIGIT expression; however, it is limited by the highly heterogeneous and dynamic expression of immune checkpoint molecules [22]. Therefore, a non-invasive method is necessary to detect and quantify tumoral TIGIT expression in the TME, which will aid in screening the population most likely to benefit from anti-TIGIT immunotherapies [23]. This approach has been performed using single-photon emission computed tomography and positron emission tomography (PET) radiotracers for CTLA-4, PD-1, PD-L1, with some showing promising results in pre-clinical models [24–26]. PET is widely used to evaluate the characteristic of new drugs in pre-clinical studies due to its non-invasive, high-resolution and timely assessments of therapeutic effect.

Antibody-based TIGIT agents for PET imaging with zirconium-89 (^89^Zr) and copper-64 (^64^Cu) have been previously reported [27]; however, due to the long half-life of monoclonal antibodies and the relatively expensive production costs of ^89^Zr and ^64^Cu, the long retention of radioactive compounds may cause potential radiation damage to the body. Therefore, it is essential to develop low molecular weight-based radioactive compounds. Wang Xb [28] et al. utilized a ^68^Ga-labeled D-peptide antagonist, ^68^Ga-GP12, was developed and validated for PET imaging of TIGIT expression, and the potential of ^68^Ga-GP12 for PET/CT imaging of TIGIT expression were also evaluated in a pilot study with advanced NSCLC patients. However, only two clinical volunteers were recruited in the clinical trial, and whether this probe can be applied in clinical need to further validation. On the other hand, the tumor uptake of GP12 (SUVmax) and the ratio of target/non-target was lower compared to ^18^F-FDG, indicating that the radiotracer needs to be further optimized for clinical transformation. ^68^Ga is one of the most commonly used positron nuclides in clinical practice, and its availability from a generator and its proper half-life of 67.6 min, which matches well with the pharmacokinetic of a peptide, make it a promising candidate for in vivo imaging [25]. An increasing number of ^68^Ga-based radiotracers including ^68^Ga-DOTATATE and ^68^Ga-PSMA have gained widespread use in clinical practice.

In this study, a novel D-peptide TBP-3, that was first reported by Zhou et al. [29], was conjugated with ^68^Ga to non-invasively detect TIGIT expression in tumor-bearing BALB/c mice. Micro-PET and biodistribution studies demonstrated that ^68^Ga-DOTA-^D^TBP-3 is a promising radiotracer for non-invasive detecting of TIGIT in the TME.

## Materials and methods

### Cell lines and culture conditions

The murine mammary cancer cell line 4T1 and human melanoma cell line A375 were purchased from Shanghai Cell Bank of Chinese Academy of Sciences (Shanghai, China) and cultured in RPMI 1640 medium supplemented with 10% fetal bovine serum (FBS) (Gibco), penicillin (100 U/ml), and streptomycin (0.1 mg/ml) (Gibco, Grand Island, NY, USA) in a T25 flasks, which was placed in a humidified incubator at 37℃ in an atmosphere containing 5% CO_2._

### Reagents and instruments

DOTA-(COOt-Bu)_3_ was purchased from Macrocylics (Plano, TX, USA). A [^68^Ge/^68^Ga] generator (ITG GmbH, Berlin, Germany) was used to elute [^68^Ga] GaCl_3_. [^68^Ga] GaCl_3_ was eluted from the generator using 5 ml of 0.1 M HCI, and then passed through a cation exchange cartridge to trap the ^68^Ga ions alone. Finally, a concentrated NaCI/HCI solution was used to obtain the trapped pure ^68^Ga_._ High-performance liquid chromatography (HPLC) and mass spectrometry were performed on LC-20AT (Shimadzu Corporation, Tokyo, Japan) and LCMS-2020 (Shimadzu Corporation, Tokyo, Japan), respectively.

### Synthesis of ^68^Ga-DOTA-^DTBP^-3

^D^TBP-3 and DOTA-^D^TBP-3 were synthesized by Shanghai Apeptide Co. (Shanghai, China). Briefly, 1 mg of DOTA-^D^TBP-3 was dissolved in 250 μL of 0.25 M sodium acetate buffer (pH 8.6). Subsequently, the pH of the peak fraction of the 1.5 mL ^68^GaCl_3_ (140–360 MBq) in 0.05 M HCI was adjusted to approximately 4.0 by adding 450 μL 0.25 M sodium acetate (pH 8.6). Then, DOTA-^D^TBP-3 (3.78–9.72 μL) was incubated with 140–360 MBq of ^68^ Ga for 10 min at 100 °C with a final pH of 4.0. The compounds were purified using a preconditional C-18 Sep-Pak cartridge column, rinsed with 10 mL of ultrapure water, and eluted with 1.0 mL of ethanol. Radio-HPLC was performed to assess the radiochemical purity of the radiotracer. A semipreparative C-18 Luna column (5, 10 × 250 mm) was used with methanol (0.1% TFA) and water (0.1% TFA) as the mobile phase, with a flow rate of 4.5 mL/min, the linear gradient from 65/35 (methanol/water) to 85/15 (methanol/water) exceeding 30 min for peak separation.

### In vitro binding assay

The equilibrium dissociation constant of ^68^Ga-DOTA-^D^TBP-3 for TIGIT was determined by a cell-saturated assay. Briefly, 1 × 10^6^ 4T1 cells/well were grown to confluence in 24-well plates. After incubating the cells for 30 min 150 μL 1% BSA/PBS, serial dilutions of ^68^Ga-DOTA-^D^TBP-3 (from 1 × 10^–4^ to 5 × 10^2^ nM) preparations were added to each well in triplicate and then co-incubated at 37℃ for 0.5 h. The wells were washed 3 times with cold PBS, and then 0.1 M NaOH solution was used to lyse the cells. The supernatant was collected, precipitated separately, and the radioactivity was measured using an automated *γ* counter. Non-specific cell binding was determined by adding 40-fold ^D^TBP-3 (about 120.0 μg), incubating for 0.5 h, and then adding the ^68^Ga-DOTA-^D^TBP-3. Equilibrium dissociation constant K_D_ was analyzed using a computer program.

For cell uptake experiments, 4T1 and A375 cells were incubated with ^68^Ga-DOTA-^D^TBP-3 at different time points (15, 30, 60 and 120 min), respectively. Then, the cells were washed 3 times with cold PBS, and 0.1 M NaOH solution was used to lyse the cells and collected for *γ*-count.

### Animal studies

All animal procedures were strictly implemented in accordance with the guidelines approved by the Institutional Animal Care and Use Committee of Wuhan University.

Female BALB/c mice of 4–5 weeks were purchased from Beijing Biotechnology Co., Ltd. (Beijing, China). The 4T1 murine cancer model was established by the injection of 3 × 10^5^ cells/150 μl into the front right limbs of female BALB/c mice. Experiments started when the tumors reached a volume of approximately 250–350 mm^3^.

### Micro-PET studies

PET images were acquired on a micro-PET scanner (InliView-3000B, Minfound, Hangzhou, China). Mice bearing subcutaneous tumors were anesthetized with isoflurane (1.0–1.5%) and then were injected with about 5.55 MBq of ^68^Ga-DOTA-^D^TBP-3 via the tail vein. In the blocking group, tumor-bearing BALB/c were pretreated with the 40-fold excess of ^D^TBP-3 for 0.5 h, and then injected equal dose of radiotracer with the above groups. Static imaging (15 min) was collected at different time points (0.5, 1 and 2 h post-injection).

### Biodistribution and pharmacokinetics studies

Female BALB/c tumor-bearing mice with subcutaneous 4T1 xenografts were randomly divided into 2 groups (*n* = 9 for the experimental group, and *n* = 3 for the blocking group), and then were injected with 3.70 MBq of ^68^Ga-DOTA-^D^TBP-3 via the tail vein. At different time points (0.5, 1 and 2 h post-injection for the experimental group, 0.5 h post-injection for the blocking group), the mice were euthanized, and the tumor, main organs (muscle, bone, large intestine, small intestine, stomach, kidney, spleen, liver, lung, heart, brain) and blood were weighed and counted by a γ counter. The results are expressed as percentage injected dose per gram of tissue (% ID/g), and the values were calculated based on signal decay correction and normalization to external ^68^Ga standards.

### HE&IHC for TIGIT expression

For IHC, harvested tumors were fixed in 4% paraformaldehyde and then embedded. After deparaffinization with xylene and an alcohol gradient, antigen retrieval was performed using 10 mM citrate buffer (pH 6.0). An anti-mouse TIGIT monoclonal antibody (catalog NO. BE0274, Bio X cell) was used as primary antibody. Tumor sections were incubated with the primary anti-mouse TIGIT antibody with a dilution of 1:500 at 4℃ overnight, washed with PBS for twice, and then incubated with the secondary antibody for 1 h at room temperature. The slides were counterstained with hematoxylin, dehydrated with an alcohol gradient, washed with xylene, and fixed with a cover slip.

### Statistical analysis

All data were analyzed with the software Graphpad Prism 8.0 (Graphpad Software Inc., San Diego, CA, USA), with *P* < *0.05* being considered statistically significant.

## Results

### Synthesis and characterization of ^68^Ga-DOTA-^DTBP^-3

**Fig. 1:**
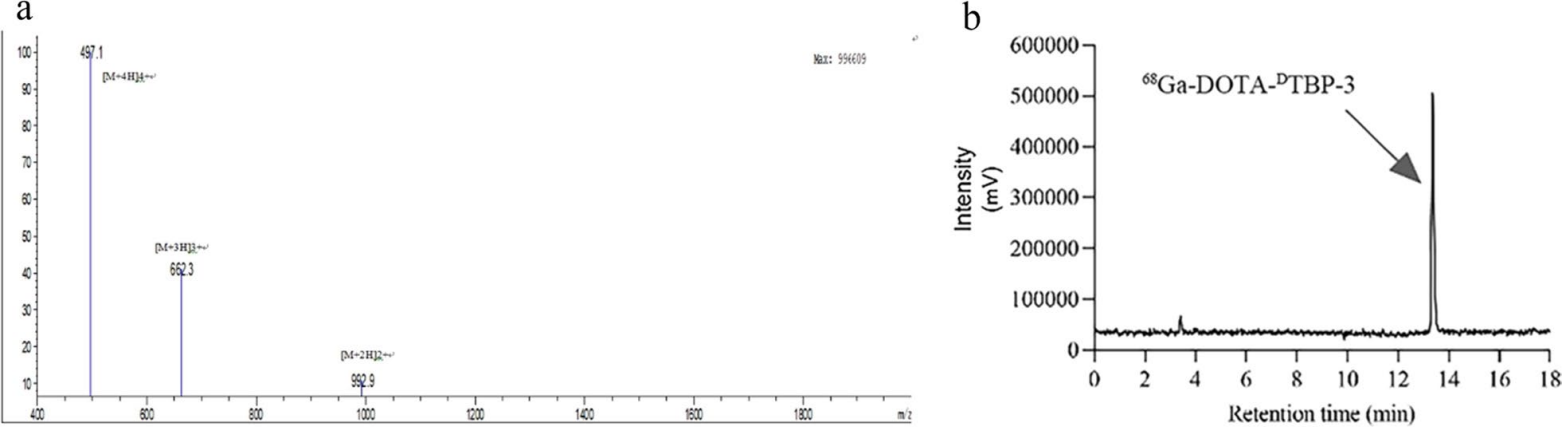
The characterization of DOTA-^D^TBP-3 was identified by mass spectrometer (**a**). The retention time of ^68^ Ga-DOTA-^D^TBP-3 was about 13.4 min identified by radio-HPLC (**b**)

The low molecular weight D-peptide TBP-3 that targets TIGIT was reported to penetrate and accumulate in the tumor tissues. To utilize this characteristic for non-invasive PET imaging of TIGIT expression, the DOTA-^D^TBP-3 was synthesized. DOTA-^D^TBP-3 was obtained with its chemical purity more than 95% (Fig. 1a). The retention time of ^68^Ga-DOTA-^D^TBP-3 was about 13.4 min (Fig. 1b). The radiochemical purity of ^68^Ga-DOTA-^D^TBP-3 in FBS was 97.23 ± 0.59% after purification, and the molar activity was calculated to be 88.34 ± 4.56 MBq/nmol. The stability of ^68^Ga-DOTA-^D^TBP-3 in FBS was demonstrated as a radiochemical purity of 89.24 ± 1.82% at 4 h in room temperature, which revealed its relative stable.

### In vitro studies

**Fig. 2:**
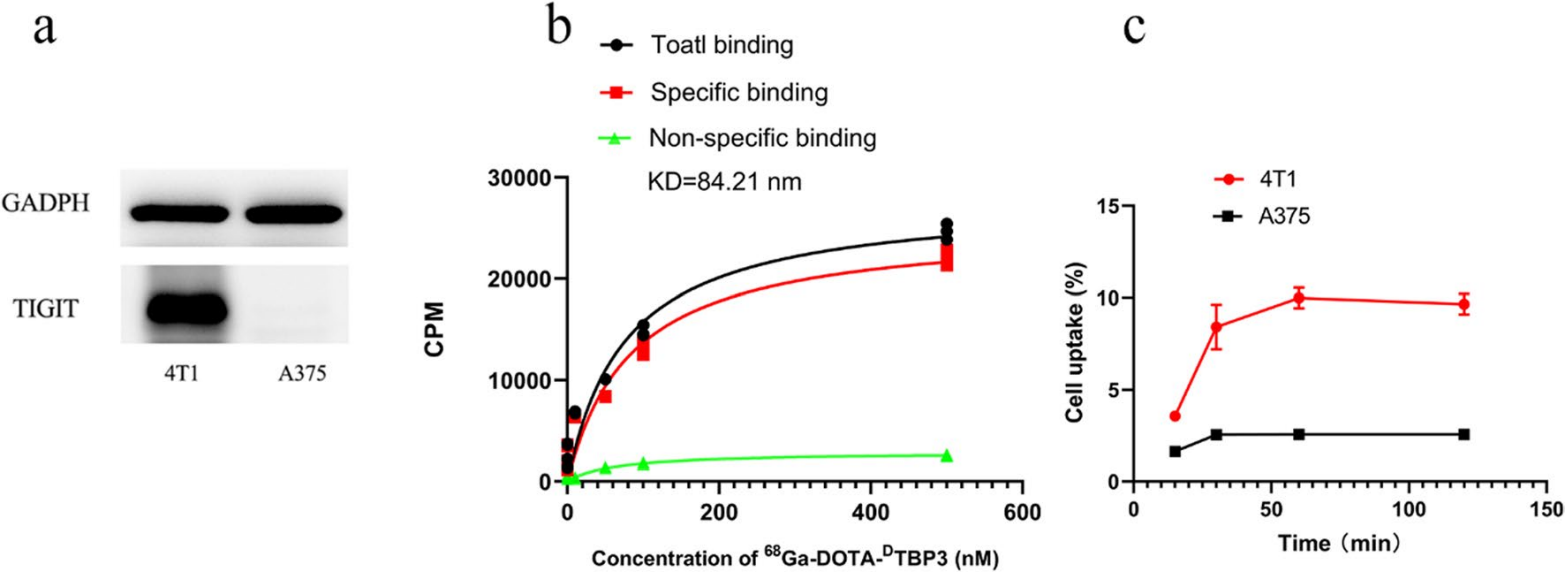
The TIGIT expression of 4T1 and A375 cells was demonstrated by western blot (**a**). Binding curve of ^68^ Ga-DOTA-^D^TBP-3 to TIGIT (**b**), showing a relative high affinity to murine TIGIT with the equilibrium dissociation constant K_D_ was 84.21 nM. Cell uptake curve of 4T1 and A375 cells with different time points (15, 30, 60 and 120 min), 4T1 and A375 cells have a highest uptake of radiotracer with 8.41 ± 1.22% and 2.56 ± 0.19% at 0.5 h, respectively (**c**)

The TIGIT expression of 4T1 and A375 cells was demonstrated by western blot (Fig. 2a), and 4T1 cell has a high TIGIT expression while A375 cell has a low TIGIT expression. A cell-saturated binding assay was performed to determine the affinity of radiotracer to murine TIGIT. The radiotracer showed a relative high affinity to murine TIGIT with the equilibrium dissociation constant K_D_ was 84.21 nM (Fig. 2b). 4T1 and A375 cells have a highest uptake of radiotracer with 8.41 ± 1.22% and 2.56 ± 0.19% at 0.5 h, respectively (Fig. 2c). The 4T1 cellular uptake of the radiotracer could be blocked by excess unlabeled ^D^TBP-3, indicating their specific binding.

### Micro-PET imaging

**Fig. 3:**
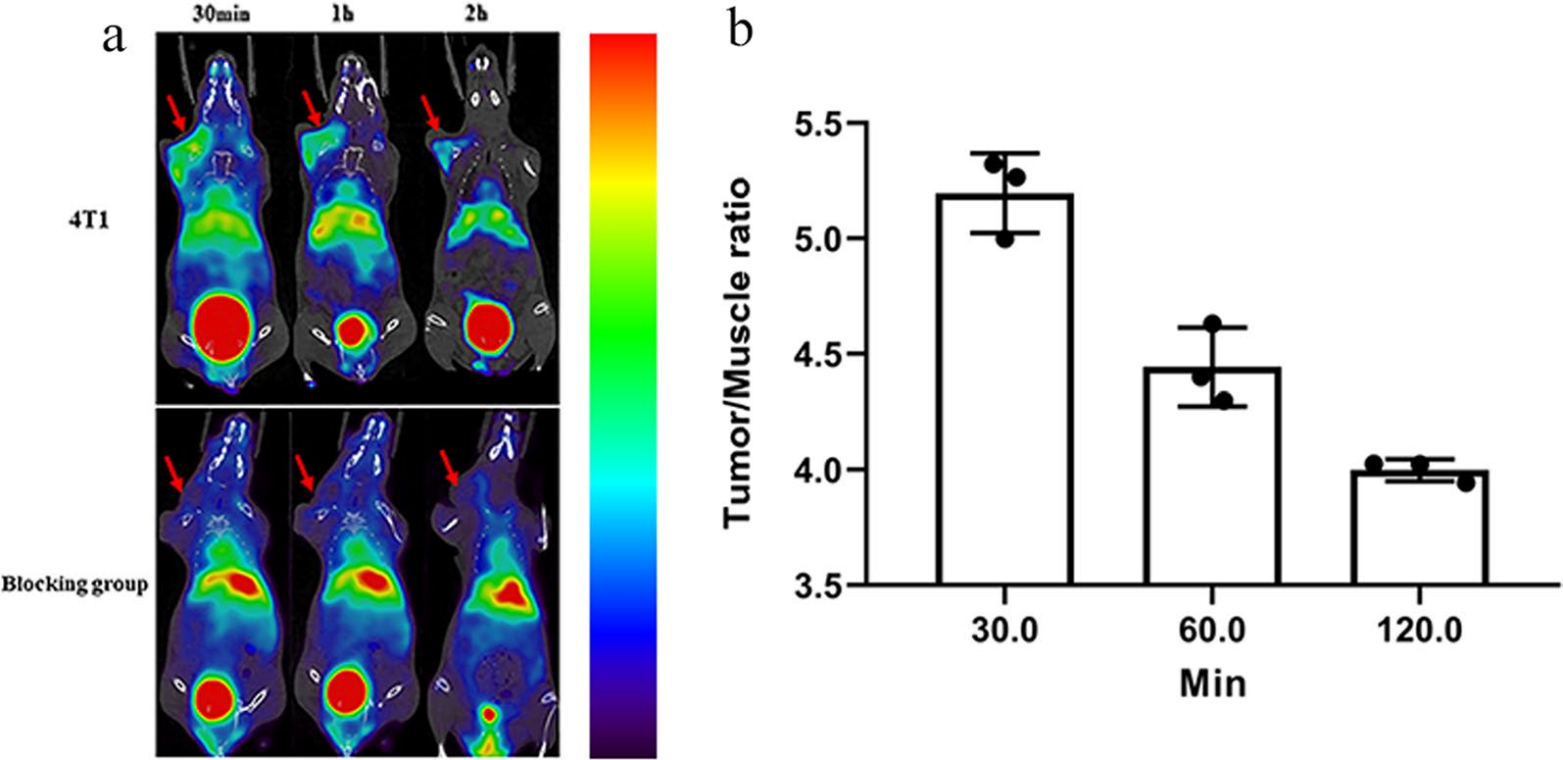
PET images of tumor-bearing mice. Static images at 0.5, 1, and 2 h post-injection of 4.44 MBq ^68^ Ga-DOTA-^D^TBP3 (*n* = 3), ^68^ Ga-DOTA-^D^TBP3 could be blocked by ^D^TBP3 at 0.5, 1 and 2 h (While the blocking group at 1, 2 h were not shown) (**a**), and tumor/muscle rations were shown in **b**

**Fig. 4:**
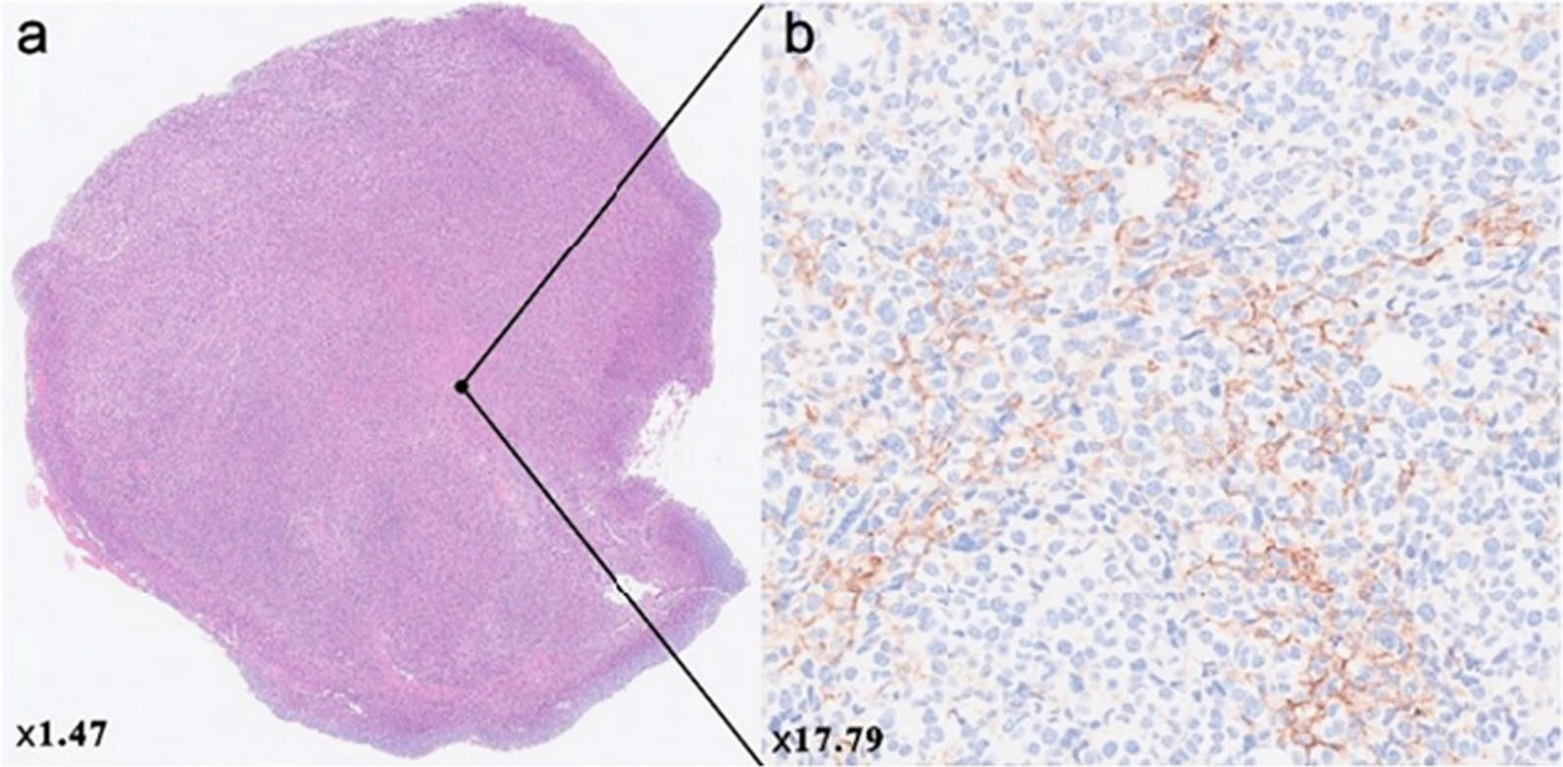
HE and IHC of 4T1 tumor (**a** and **b**)

The TIGIT-targeting ability of ^68^Ga-DOTA-^D^TBP-3 was performed on micro-PET in 4T1 tumor-bearing mice (Fig. 3a). As shown in Fig. 3a, 4T1 tumor-bearing BALB/c mice showed rapid and high uptake (3.49 ± 0.14) of ^68^Ga-DOTA-^D^TBP-3 in the tumors at 0.5 h following intravenously injection, with the highest tumor/muscle ratio of 5.20 ± 0.16. There was no obvious uptake of ^68^Ga-DOTA-^D^TBP-3 by tumors in 4T1 tumor-bearing mice pretreated with the unlabeled ^D^TBP-3. This result was further confirmed by HE and IHC (Fig. 4a and b).

### Ex vivo studies

**Fig. 5:**
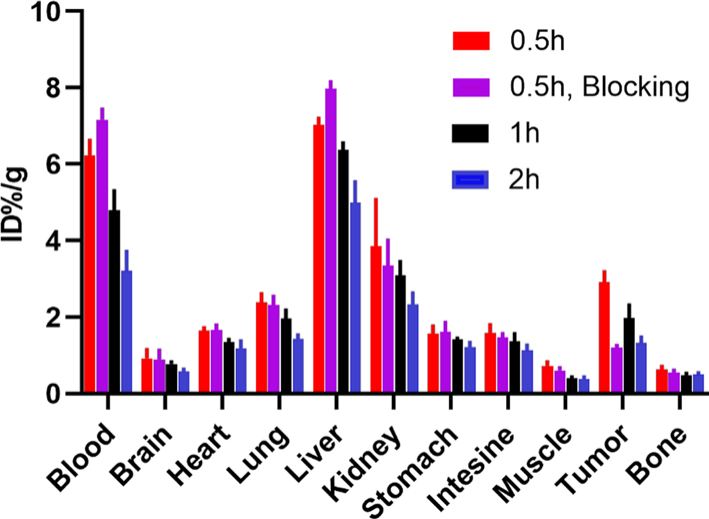
Biodistribution analysis at 0.5, 1 and 2 h post-injection of ^68^ Ga-DOTA-^D^TBP3 in 4T1 tumor-bearing mice model (*n* = 3) (Fig. 4)

The biodistribution data of ^68^Ga-DOTA-^D^TBP-3 in 4T1 tumor-bearing BALB/c mice are presented in Fig. 5. At 0.5 h post-injection, the kidney had relatively high radiotracer uptake, while the liver and remaining organs showed low radiotracer uptake. The biodistribution results indicated that ^68^Ga-DOTA-^D^TBP-3 could be quickly cleared from the blood, which resulted in a high tumor-to-muscle ratio and a high tumor-to-blood ratio at 0.5 h post-injection in 4T1 tumor-bearing mice.

## Discussion

TIGIT is expected as a promising therapeutic target for the next generation of immune checkpoints, and the efficacy of anti-TIGIT immunotherapy is correlated with the expression of TIGIT in the TME. However, detection by IHC relies on samples taken by biopsy, which is invasive and does not provide a comprehensive quantification of expression levels in the whole body. PET is a non-invasive imaging technology that can be performed in real time, is repeatable, and allows dynamic detection of all lesions [27]. Thus, it could greatly facilitate the screening of patients who may benefit from specific immunotherapies, evaluations of treatment efficacy, and treatment adjustments. In this study, a novel D-peptide TBP-3, was conjugated with ^68^ Ga to non-invasively detect TIGIT expression in tumor-bearing BALB/c mice.

PET has been widely used to image other immune checkpoints proteins including PD-1/PD-L1 [30–32], LAG-3 [33], and TIM-3 [34] with radionuclide-labeled antibodies. Apart from the shortcomings of immune-related side effects, poor tissue infiltration is feature of antibody themselves; additionally, their relatively long half-lives means than imaging can only be performed 4–7 d to allow for better tissue contrast [35]. In addition, low molecular weight peptide drugs are ideal candidates for PET imaging because they have the appropriate half-lives and strong tissue infiltration. High-affinity peptides targeting PD-L1, especially TPP-1 and WL12 have been widely used for PET imaging with various radionuclide label, includes ^68^Ga, ^18^F, ^64^Cu and ^89^Zr [25, 36–42]. PET imaging of TIGIT has been explored using ^64^Cu and ^89^Zr-labeled TIGIT antibody and remains to be further developed. Here, we conducted PET imaging with ^D^TBP-3, which is a high-affinity D-peptide for TIGIT with proteolysis-resistance and strong tissue infiltration [29]. As to the radionuclide label, ^68^Ga was selected. ^68^Ga is widely used in clinical centers and has the benefit of low radiation exposure. More importantly, the half-life of ^68^Ga has comparative pharmacokinetic properties with peptides [25].

For PET imaging of TIGIT, a murine breast cancer cell line with high expression of TIGIT was used [43]. As expected, BALB/c tumor-bearing mice had the highest uptake of PET radiotracer at 0.5 h, and this could be blocked by excess unlabeled ^D^TBP-3, which demonstrated that ^68^Ga-DOTA-^D^TBP-3 could specifically bind to TIGIT in vivo. Fortunately, Wang Xb [28] et al. also utilized a ^68^Ga-labeled D-peptide antagonist, ^68^Ga-GP12, was developed and validated for PET imaging of TIGIT expression, and the potential of ^68^Ga-GP12 for PET/CT imaging of TIGIT expression were also evaluated in a pilot study with advanced NSCLC patients. Although the same D-peptide was used in both studies, there were still some differences of the probe. They modified the peptide with polyethylene glycol and then coupled with NOTA while our study directly used DOTA to couple the D-peptide. The affinity of ^68^Ga-GP12 to TIGIT and the stability of ^68^Ga-GP12 in FBS were better than ^68^Ga-DOTA-^D^TBP-3 may attribute to the following reasons. Firstly, the structure of the NOTA ring matches ^68^Ga better than DOTA; secondly, PEG modified peptide increases the water solubility of the probe. In the experiment, we found that the half-life of ^68^Ga-DOTA-^D^TBP-3 was fast in vivo, and the peak uptake of tumor tissue was about 0.5 h which was quickly than them. On the other hand, liver uptake was obvious lower than our study which due to the PEG modified peptide. In addition, there are some limitations of this study. TIGIT is mainly expressed in tumor infiltrating lymphocytes, while a cell line with tumor cells overexpressed TIGIT was used in our study which may not reflect the true situation of the TME. On the other hand, we did not compare it with ^18^-F-FDG.

Single domain antibody is particularly suitable for molecular imaging due to its small molecular weight and high affinity with receptor, and ^68^Ga-labeled PD-L1 single domain antibody showed better pharmacokinetic in pre-clinical or clinical practice. In the future, NOTA conjugated TIGIT single domain antibody would be used to monitor tumor TIGIT expression, and human TIGIT would be explored for further study.

## Conclusions

This work validates the TIGIT-specific peptide for quantification of the expression level of TIGIT in the TME. We demonstrate that ^68^Ga-DOTA-^D^TBP-3 could serve as a specific molecular probe for detecting TIGIT expression in vivo. ^68^Ga-DOTA-^D^TBP-3 was evaluated in BALB/C mice with 4T1 breast cancer and showed the potential to quantitatively detect TIGIT expression from 0.5 min to 1 h post-injection. These results indicate ^68^Ga-DOTA- ^D^TBP-3 as a potential companion diagnosis or evidence for patient stratification of TIGIT blocking-based cancer immunotherapy.

## Data Availability

All data generated or analyzed during the current study are included in this published article.
